# The Prevalence of Polycystic Ovary Syndrome (PCOS) in High School Students in Rasht in 2009 According to NIH Criteria 

**Published:** 2011-02-20

**Authors:** Maryam Asgharnia, Fariba Mirblook, Mitra Ahmad Soltani

**Affiliations:** Department of Obstetric and Gynecology, Guilan University of Medical Sciences, Research Vice Chancellorship, Rasht, Iran

**Keywords:** Polycystic Ovary Syndrome, Hirsutism, Acne, Male Pattern Baldness, Oligomenorrhea, Amenorrhea

## Abstract

**Background:**

Polycystic ovary syndrome (PCOS) is the most common endocrine disorder in women associated with many reproductive, endocrine, metabolic and cardiovascular dysfunctions. This study aimed to determine the prevalence of PCOS among high school students in Rasht.

**Materials and Methods:**

In a cross–sectional study, 1850 students were selected by a multi-stage cluster sampling from all high schools in Rasht. The inclusion criteria were: age 17-18 years, menarche from 10-16 years, normal prolactin and thyroid stimulating hormone (TSH) values, no history of anatomical malformation, no use of medication or hair-removal techniques, and a history of oligo- or amenorrhea. PCOS was diagnosed if both menstrual dysfunction and clinical hyperandrogenism were detected.

**Results:**

Mean age of subjects was 17.2 ± 0.7 years and the age of menarche was 12.8 ± 0.9 years. Of all students, 378 (20.4%) had oligomenorrhea and PCOS was diagnosed in 210 (11.34 %) according to the National Institute of Health (NIH) definition. PCOS subjects, mean body mass index (BMI), waist circumference, and waist/hip (W/H) ratio were 21.1 ± 3.6, 73.4 ± 8.0 cm and 0.77 ± 0.05, respectively. A family history of diabetes mellitus type 2 was reported in 24.7% of subjects.

**Conclusion:**

The prevalence of PCOS in this study was similar to the international estimates of 10-20% in Caucasians. A long-term follow-up is needed to compare the accuracy of clinical determination of the disease versus diagnosis based on hormonal and/or sonographic assessments.

## Introduction

Polycystic Ovary Syndrome (PCOS) is the most common endocrinopathy in women and the most common cause of anovulatory infertility, affecting 5-10% of the female population (1).

According to Ojaneimi,PCOS in teenagers is characterized by irregular menstrual cycles (generally less than six menses per year) and clinical or biochemical features of hyperandrogenism (2).

PCOS typically presents during adolescence and is a heterogeneous syndrome classically characterized by features of anovulation (amenorrhoea, oligomenorrhoea, irregular cycles) combined with symptoms of androgen excess (hirsutism, acne and alopecia).

Hyperandrogenism, most particularly in women with PCOS, is a diagnosis of androgen levels that does not virilize, yet is above normal limits (3).

Some rely on the clinical presentation of peripheral androgen excess in women to make the diagnosis of hyperandrogenism as part of the PCOS phenotype that includes midline hirsutism, acne and androgenic alopecia (4-6). This syndrome is a common problem affecting approximately 5% of women of reproductive age when defined by the clinical features of anovulation and hyperandrogenism (7).

The old National Institute of Health (NIH) criteria included both oligo-amenorrhoea and chronic anovulation in addition to the presence of either clinical or biochemical hyperandrogenism. The Rotterdam 2004 Consensus Workshop has proposed that PCOS is a syndrome of ovarian dysfunction and recommended that two of the following criteria should be present in order to establish a diagnosis: chronic oligo- or anovulation for more than six months, clinical and/or biochemical evidence of hyperandrogenism and polycystic ovaries on ultrasound (8).

The present study is to determine the prevalence of clinical PCOS in high school students in Rasht in 2009 according to NIH criteria.

## Materials and Methods

In a cross-sectional study, 1850 students were selected by a multi-stage cluster sampling from all high schools in Rasht. The inclusion criteria were: age 17-18 years, menarche of 10-16 years, normal prolactin and thyroid stimulating hormone (TSH) values, no history of gross anatomical malformation (e.g., imperforated hymen) as evidenced by physical examination or cosmetic hair removal, and a history of oligoor amenorrhea. Clinical PCOS was diagnosed if both menstrual dysfunction and clinical hyperandrogenism were detected following history and physical examination. The diagnoses of hypothyroidism and hyperprolactinemia were excluded by normal TSH and prolactin. Nonclassic adrenal hyperplasia was excluded by familial history.

The study was approved by the Department of Ethics at Guilan University of Medical Sciences. Approved written informed consents and personal medical histories were obtained from each student according to a validated questionnaire that consisted of three parts: history, Ferriman-Gallway scoring system (9) and physical examination.

Students who used medication, hair-removal techniques and had a previous history of disease were excluded from the study.

Oligomenorrhea was defined as irregular menstrual cycles longer than 35 days or a history of nine or fewer menses annually. Amenorrhea was defined as a lack of menstrual bleeding for three consecutive months during the previous year.

Clinical hyperandrogenism was defined by a Ferriman-Gallway score of 6 or more (4) based on male pattern baldness, severe or persistent acne characterized by inflamed papules, pustules and superficial or pus-filled cysts and deep inflamed nodules, or refractoriness to therapy (9). For each student, the physical examination was performed by a physician; weight and height were measured while the subjects were lightly clothed and barefooted. Body mass index (BMI) was calculated (10). A score 6 or greater seemed more indicative since hirsutism has a slow progression in adolescence and obesity was defined as a BMI of ≥ 25 (9). Waist circumference was measured midway between the top of the iliac crest and the lower rib margin. A waist-to-hip (W/H) ratio greater than 0.85 indicated android fat distribution (11).

According to the history and physical examination, clinical PCOS was diagnosed in students who presented with both menstrual dysfunction and clinical hyperandrogenism.

## Results

The results are presented as mean ± standard deviation. The mean age of the students was 17.2 ± 0.7 years and mean age at menarche was12.8 ± 0.9 years.

**Fig 1:**
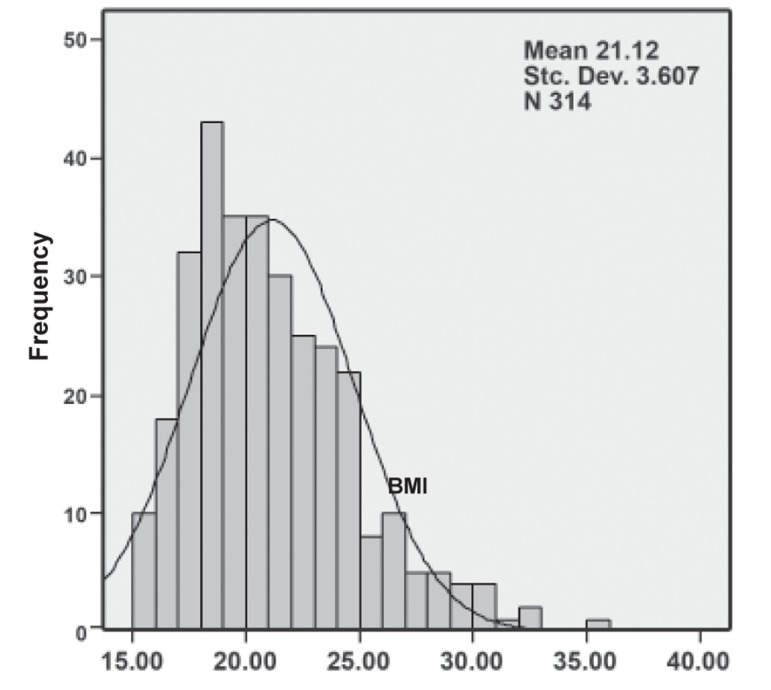
Histogram of BMI distribution in subjects. The distribution is skewed to the left, yet it is not statistically significant.

**Fig 2:**
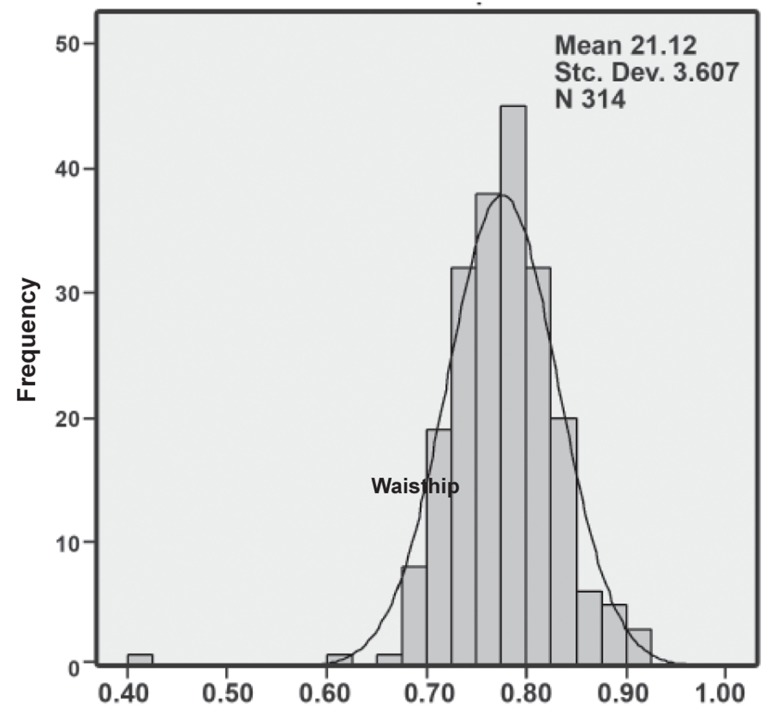
Histogram of the waist/hip distribution in subjects. The distribution is skewed to the right, yet it is not statistically significant.

Three hundred seventy eight girls (20.4%) had oligo- or amenorrhea. PCOS was diagnosed in 210 (55.6% of 378 or 11.34% of the total 1850) based on two or three out of the three criteria of PCOS according to the NIH definition, with hirsutism in 91 (24.1%) and severe acne in 101 (26.7%) cases. In PCOS subjects, the mean BMI, waist circumference and W/H ratio were 21.1 ± 3.6 (Fig 1), 73.4 ± 8.0 cm and 0.77 ± 0.05 (Fig 2), respectively. A family history of diabetes mellitus type 2 was reported in 24.7% of subjects.

## Discussion

PCOS is a chronic condition with manifestations that most commonly occur in adolescence with oligomenorrhoea or amenorrhoea and transition over time into problems that include infertility and metabolic complications (12).

The occurrence of PCOS in the sample of high school girls studied in Rasht was 11.34%. However, the incidence of PCOS among adolescents in another study in the same region that utilized ultrasonography was estimated to be 11% (13).

Screening of an unselected population in the Southwestern United States showed an incidence of 4% (4.7% in white and 3.4% in black women) (14).

Diamanti-Kandaraskis et al. (15) reported a 6.8% prevalence for PCOS on the Greek island of Lesbos. The prevalence was 6.5% in Caucasian women in Madrid, Spain (16). The higher rate of PCOS in this study can be attributed to the fact that we used NIH rather than Rotterdam criteria.

In one study PCOS was found in 9% of girls who had regular menstrual cycles, 28% in those with irregular menstrual cycles and 45% in those who were oligomenorrheic. It has been concluded that PCOS in adolescents is similar to adults and that it is clearly associated with menstrual dysfunction and/or high androgen and LH levels, but the relationship with hirsutism and acne is less clear (17). It should be noted that hormonal evaluation and ovarian sonography were not performed in our subjects based on the statements that clinical examination is more diagnostic than polycystic-appearing ovaries as seen with sonography in the adolescent population (10, 14) .

Moreover, some women with PCOS do not display polycystic ovary (PCO) morphology on sonography. There may be other ovarian morphologies such as hyperthecosis (18) or discordant ovaries of varying morphology (19).

In our study, 20.4% of students had menstrual dysfunction. As noted by other investigators, oligomenorrhea, even in the absence of hirsutism or acne, seems to be associated with a subtle or overt elevation in serum androgens and may present a discrete form of PCOS (20).

Van Hooff et al. have reported that oligomenorrhea in adolescents is not a stage in the physiological maturation of the hypothalamic- pituitary-ovarian axis, but an early sign of PCOS associated with subfertility (21).

Thus early detection of the syndrome based on clinical findings (mainly oligo- or amenorrhea) offers an opportunity for early intervention to prevent or limit the impact of cutaneous and reproductive symptoms, and the longer-term effects of metabolic disturbances (22).

In our study PCOS patients had a mean BMI of 21.2 ± 3.6, waist circumference of 73.4 ± 8 cm and mean W/H ratio of 0.77 ± 0.05. Thus, our patients were not overweight or obese. As suggested by some authors, the risk of PCOS is minimally increased with obesity. PCOS is a genetically determined ovarian disorder characterized by excessive androgen production and the heterogeneity of PCOS can be explained by the interaction of this disorder with environmental factors such as diet and obesity (23-25).

## Conclusion

The prevalence of clinical PCOS in this study was similar to international estimates of 10-20% in Caucasians. A long-term follow-up is needed to compare the accuracy of clinical determination of the disease versus diagnosis based on hormonal and/or sonographic assessment. The prevalence of clinical PCOS can be estimated using Rotterdam criteria which despite some pitfalls can be more defining than NIH criteria.
